# Modeling student satisfaction in online learning using random forest

**DOI:** 10.1038/s41598-025-06686-3

**Published:** 2025-07-02

**Authors:** Jinlei Li, Xiaowei Chen

**Affiliations:** 1 Academic Affairs Office, Zhejiang Institute of Communications, Hangzhou, China; 2 College of Art, Zhejiang Shuren University, Hangzhou, China

**Keywords:** Online learning platforms, Student satisfaction, Random forest, Psychological well-being, Emotional stability, Cognitive engagement, SMOTE, Nonlinear modeling, Statistics, Human behaviour

## Abstract

The rapid expansion of online education has intensified the need to investigate the multifactorial determinants of university students’ satisfaction with digital learning platforms. While prior studies have often examined technical or pedagogical components in isolation, limited attention has been paid to their interaction with students’ psychological well-being, particularly through nonlinear mechanisms. To address this gap, this study employs a Random Forest–based framework to model satisfaction using a multidimensional dataset from 782 university students. Measured variables included platform usability, content quality, emotional experience, and self-regulation. Data were standardized via Z-scores, and class imbalance was addressed using the Synthetic Minority Over-sampling Technique (SMOTE). Model performance was evaluated using accuracy, F1-score, and area under the ROC curve (AUC). Results identified platform stability and content update frequency as the most influential predictors, with AUC values exceeding 0.95 across most satisfaction levels. Psychological factors—especially perceived enjoyment and emotional stability—also contributed significantly. Partial dependence plots revealed threshold and saturation effects, highlighting complex nonlinear patterns missed by traditional linear models. However, performance declined in predicting low-satisfaction cases (AUC = 0.70), likely due to subgroup underrepresentation. This study contributes theoretically by integrating cognitive-affective dimensions, methodologically by demonstrating the utility of machine learning in modeling nonlinear interactions, and practically by providing actionable insights for platform optimization. Future research should incorporate additional psychological constructs, such as cognitive load and resilience, and apply the model across more diverse learner populations to enhance generalizability and support inclusive, user-centered digital education.

**Supplementary Information:**

The online version contains supplementary material available at 10.1038/s41598-025-06686-3.

## Introduction

User Satisfaction has long been a proxy for platform effectiveness and user retention within information systems and educational research. Foundational models such as EUCS^1^ and TAM^2^ provided the theoretical basis, which has since been extended to account for the cognitive-affective complexity of digital learning environments. For instance, emotional and behavioral constructs have been incorporated into TAM for analyzing MOOC engagement, while an extended TAM-ISCM model integrating perceived enjoyment, trust, and well-being was proposed to explain continuance intention on innovative learning platforms^4^. Similarly, a validated, multidimensional satisfaction model aligned with EUCS dimensions was developed for LMS evaluation^5^.

However, empirical implementations of these models remain predominantly linear, limiting their ability to capture nonlinear interactions and latent psychological dynamics^6,7^. These limitations are particularly salient in contemporary learning environments, where satisfaction often arises from complex interdependencies between system characteristics and user traits. Moreover, traditional satisfaction models have been critiqued for insufficiently addressing psychological and emotional factors. Emotional regulation, subjective well-being, and resilience have been empirically linked to satisfaction, academic achievement, and digital persistence^8,9^. The COVID-19 pandemic has further underscored the importance of these factors, exposing disparities in students’ emotional readiness, engagement capacity, and equitable access to digital resources^7,10^.

Despite growing scholarly interest in student satisfaction, much of the existing literature continues to adopt a reductionist lens—focusing primarily on technical dimensions such as interface design or content availability—while overlooking the dynamic interplay between platform functionality and learners’ cognitive-affective states^8,11^. Such an approach risks oversimplifying the complex, context-dependent nature of satisfaction formation. To overcome these limitations, recent research has increasingly turned to machine learning (ML) methods, which provide flexible, data-driven alternatives to traditional linear modeling. Ensemble-based algorithms such as Random Forest (RF) have demonstrated notable efficacy in detecting nonlinear interactions and complex variable dependencies within educational datasets^12,13^. In contrast to models that presume independent and additive effects, RF can model higher-order interactions and generate robust feature importance metrics^9,14^. Applications of RF in educational research have included predicting student attrition^15^, personalizing content recommendations^16^, and evaluating satisfaction in multimedia learning contexts^3^, further affirming its methodological value.

Building upon these methodological advancements, the present study introduces a Random Forest–based framework for modeling university students’ satisfaction with online learning platforms. The model integrates conventional system indicators—usability, content quality, and system responsiveness—with psychological variables, including emotional stability, self-regulation, and resilience. By capturing the nonlinear, interactive effects of technological and psychological dimensions, the framework offers a more comprehensive understanding of satisfaction formation. This research contributes to the literature in three significant ways. First, it introduces methodological innovation by demonstrating the utility of Random Forest in modeling satisfaction processes that elude traditional linear frameworks. Second, it provides empirical evidence regarding the relative predictive value of platform features and psychological traits. Third, it bridges theoretical models such as TAM and ECM with contemporary machine learning approaches, advancing an integrative framework for understanding user satisfaction in digital education. To systematically investigate the unresolved issues identified in prior literature, this study formulates the following research questions:

- RQ1: Which platform-related and psychological variables influence university students’ satisfaction with online learning platforms?
- RQ2: How do these factors interact through nonlinear dynamics that elude detection by conventional linear modeling techniques?
- RQ3: To what extent can a Random Forest model accurately classify student satisfaction levels, particularly under conditions of class imbalance?

The remainder of this article is structured as follows: Sect. "Literature review" reviews theoretical and empirical perspectives on satisfaction in online learning contexts. Section "Methodology" outlines the research methodology, including data collection, preprocessing procedures, and model implementation. Section "Results" presents the empirical findings derived from the Random Forest analysis. Section "Discussion" discusses theoretical implications and practical applications, while Sect. "Conclusion" concludes with limitations and recommendations for future research.

## Literature review

### Research on student satisfaction with online learning platforms

The accelerated growth of digital education has prompted extensive inquiry into the determinants of student satisfaction in online learning environments. Contemporary studies increasingly emphasize that satisfaction arises from a complex interplay of technical, pedagogical, and affective dimensions—including system functionality, content accessibility, emotional experience, and interaction design^3,13,17,18^. A substantial body of empirical work has identified specific predictors such as system responsiveness, content relevance, instructor presence, perceived platform security, and interface usability as central to shaping students’ perceptions and sustaining their engagement with digital platforms^3,17,18^. For example, the mediating role of initial trust and psychological distance in influencing satisfaction within learning management systems (LMS) has been underscored^17^, while the importance of intuitive navigation and regular content updates for fostering continuance intention has been highlighted^18^. Complementing these insights, it has been demonstrated that flexible content delivery and active teaching staff involvement significantly contribute to perceived platform usefulness and long-term learner retention^19^.

However, despite these valuable contributions, most existing models adopt linear analytical paradigms—primarily regression analysis and structural equation modeling (SEM)—which rest on variable independence and additivity assumptions. These assumptions overlook the inherently dynamic and interconnected nature of user experiences in digital learning ecosystems. Online learners are not homogeneous; they exhibit considerable variability in demographic background, cognitive strategies, emotional regulation capacities, and behavioral tendencies. A growing body of research has highlighted the significant impact of psychological attributes—such as emotional stability^15^, perceived stress^7^, self-regulation, and resilience^8,9^—on students’ evaluation of and commitment to digital learning platforms.

Traditional linear models treat these psychological and system-related variables as isolated predictors rather than interactive forces operating within complex learning ecologies. Such reductionism risks underestimating the mediating roles of psychological traits in modulating learners’ responses to technological environments, particularly during COVID-19, which has amplified concerns surrounding student mental health and well-being. Consequently, conventional satisfaction frameworks are ill-equipped to detect nonlinear effects, threshold conditions, and saturation dynamics that emerge from the interactions between system features and psychological dispositions.

This methodological constraint underscores the necessity of adopting more flexible, data-driven modeling approaches that can accommodate complex interaction patterns. Advanced machine learning techniques offer a promising alternative. They can unveil latent structures and nonlinear dependencies that traditional models will likely miss. In doing so, they may provide a more nuanced understanding of how digital platforms can be optimized to meet diverse learner needs in evolving educational landscapes.

### Psychological well-being and emotional stability

Psychological attributes—emotional stability, subjective well-being, self-regulation, and perceived self-efficacy—have increasingly been recognized as core determinants of student satisfaction in online education. Empirical research demonstrates that students with stronger emotional regulation and cognitive control are likelier to sustain engagement and report favorable evaluations of digital learning platforms^20,21^. In this regard, psychological well-being is a learning outcome and a critical interpretive lens through which learners assess key system dimensions, including content quality, interface usability, and instructional design.

These associations gained heightened relevance during the COVID-19 pandemic, which amplified emotional disparities among learners. Studies have shown that students with greater emotional resilience and coping capacity were better able to navigate the challenges of remote learning, resulting in higher satisfaction and more persistent use of digital platforms^7,8^. This period underscored the imperative of integrating affective dimensions into satisfaction modeling, particularly under conditions of uncertainty and psychological strain.

Reflecting this shift, recent modeling approaches have begun to embed cognitive-affective variables into predictive frameworks. Kanetaki et al. (2021)^22^, for example, a hybrid metamodel combining Generalized Linear Models (GLAR) with Artificial Neural Networks (ANN) was developed, selecting key psychological predictors via correlation analysis^22^. Their results affirmed that constructs such as perceived enjoyment and mental presence were significant predictors of satisfaction, offering empirical validation for including emotion-linked features in digital learning models. These findings point to a broader methodological transition—from technocentric paradigms to integrative models capable of capturing complex, nonlinear interactions between affective states and platform features.

Nevertheless, classical models such as the Technology Acceptance Model (TAM) and the End-User Computing Satisfaction (EUCS) framework continue to fall short in accounting for this psychological complexity. By portraying users as rational actors responding to isolated system attributes, these frameworks overlook the dynamic interplay between affective engagement and system functionality. Addressing this theoretical and methodological limitation calls for adaptive, psychologically grounded modeling strategies that more accurately represent the heterogeneous experiences of digital learners.

### Machine learning approaches in satisfaction modeling

As student satisfaction in digital learning environments is increasingly understood to arise from complex, nonlinear interactions between technological features and psychological factors, machine learning (ML) has gained prominence as a powerful analytical approach. In contrast to conventional linear models, which often assume independent and additive relationships among predictors, ML techniques, particularly ensemble algorithms such as Random Forest (RF), are well-equipped to uncover high-dimensional, context-dependent dependencies that reflect real-world complexity^12,13^.

Random Forest, a non-parametric and relatively interpretable algorithm, has demonstrated considerable utility across various educational domains. Prior applications include the prediction of course satisfaction^9^, the assessment of teaching effectiveness^23^, and the identification of students at risk of dropout^15^. Compared to deep neural networks (DNNs)—which typically require large datasets and often operate as “black boxes”—RF offers a more favorable balance between predictive accuracy and interpretability. Moreover, its robustness to multicollinearity and tolerance for small or noisy datasets make it especially suitable for educational research, where sample variability and feature overlap are common^6,24^.

One of RF’s key advantages lies in its capacity to model subtle, nonlinear interactions among psychological, behavioral, and system-level variables. For example, it was found that RF outperformed other classifiers, such as support vector machines (SVMs) and artificial neural networks (ANNs), in predicting satisfaction based on factors like perceived employability and institutional support^14^. RF has also been leveraged to explore how motivational profiles and academic self-efficacy interact with system design features, revealing nuanced patterns of student engagement^13^. Importantly, RF is a predictive tool and a heuristic instrument: its variable importance metrics can be used to identify latent constructs, refine conceptual taxonomies, and inform theoretical development^7^.

In conclusion, Random Forest represents a methodologically robust and conceptually flexible framework for modeling student satisfaction in online learning settings. Its capacity to integrate cognitive-affective traits with technological indicators, while preserving model transparency and adaptability, makes it an indispensable tool for researchers aiming to uncover hidden structures and design evidence-based improvements in digital education.

### Addressing class imbalance in satisfaction data

A recurring methodological challenge in student satisfaction modeling is the disproportionate underrepresentation of low-satisfaction responses. This class imbalance can lead to skewed model performance, inflated accuracy metrics, and reduced generalizability, ultimately compromising the interpretability and fairness of analytical results. To mitigate these issues, the Synthetic Minority Over-sampling Technique (SMOTE) has become a widely adopted solution. By synthetically generating new instances for the minority class, SMOTE rebalances the dataset and enhances the model’s capacity to detect dissatisfaction-related patterns, particularly when integrated with ensemble algorithms such as Random Forest^6,24^.

The limitations of traditional modeling frameworks in handling class imbalance have been increasingly highlighted in recent literature. For instance, it was showed that combining SMOTE with ensemble methods such as Bagging and Random Forest significantly improved F1-scores and recall in predicting student performance outcomes^25^. Similarly, SMOTE has been applied to deep learning–based educational assessments, resulting in more balanced classification performance across satisfaction tiers^4^. Extending these findings, the efficacy of a hybrid SMOTE + ENN (Edited Nearest Neighbors) approach in modeling comfort perceptions in educational spaces has been demonstrated, offering methodological insights transferable to satisfaction research^26^.

Significantly, SMOTE’s applicability extends beyond educational contexts. In psychological domains, SMOTE has been utilized to predict romantic relationship dissolution^27^, while the technique has also been employed to enhance the accuracy of depression onset forecasts among elderly populations^28^. These diverse applications highlight SMOTE’s broader relevance in domains where minority-class sensitivity is statistically and substantively critical.

These advancements underscore the value of SMOTE and its variants as indispensable tools for managing class imbalance. By improving sensitivity to underrepresented responses and preserving the integrity of analytic signals, SMOTE enables the development of more equitable, robust, and generalizable satisfaction models—particularly in settings where minority-class insights hold significant theoretical and practical implications.

### Research gaps

Despite the foundational contributions of traditional frameworks such as TAM, EUCS, and ECM, their frequent operationalization via linear models limits their ability to capture psychological complexity and nonlinear dynamics. Although prior studies have identified key satisfaction determinants—such as content delivery, system usability, and instructional effectiveness—these factors are often examined in isolation, neglecting the interactive, multifactorial nature of satisfaction formation in digital learning environments.

This reductionist lens is particularly inadequate in online contexts, where satisfaction emerges from the dynamic interplay between technological affordances (e.g., interface responsiveness, content update frequency) and learner-specific psychological traits (e.g., emotional stability, academic self-efficacy, stress resilience). Traditional models frequently overlook cross-level dependencies, resulting in an incomplete understanding of how students experience and evaluate digital learning platforms.

Furthermore, a persistent methodological limitation is the underrepresentation of low-satisfaction cases, which biases predictive accuracy and weakens generalizability. Class imbalance is rarely addressed in satisfaction modeling, leaving a gap in the fair representation of dissatisfaction signals.

To overcome these challenges, this study proposes a machine learning–based framework grounded in the Random Forest (RF) algorithm. The approach integrates system-level and psychological variables, accommodates nonlinear dependencies, and applies the Synthetic Minority Over-sampling Technique (SMOTE) to address data imbalance. Unlike post hoc regression-based diagnostics, RF enables simultaneous prediction and feature importance ranking, allowing the identification of high-impact predictors across diverse learner profiles.

This study distinguishes itself by combining psychological and technological predictors within a unified nonlinear model and systematically correcting class imbalance. These dual innovations—rarely addressed in tandem—enable a more comprehensive, equitable, and context-sensitive modeling of student satisfaction. The findings contribute to theory and practice by offering actionable insights for enhancing user-centered design and learner support in online education.

## Methodology

**Fig. 1:**
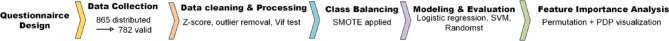
Methodology of the study.

This study employed a quantitative, data-driven approach to investigate university students’ satisfaction with online learning platforms by integrating structured questionnaire responses with machine learning–based predictive analytics. In light of the limitations inherent in conventional linear statistical methods—particularly their inability to capture complex, interactive relationships among educational variables^7,29^—Random Forest (RF) was selected as the core modeling technique due to its capability to handle nonlinear interactions and evaluate feature importance. The research design comprised four principal stages: instrument development, data collection, data preprocessing, and model construction and evaluation. The overall methodology employed in this study is illustrated in Fig. 1.

### Instrument development and data collection

865 responses were initially collected through stratified sampling across more than 20 vocational colleges in China. Institutions were selected based on geographic diversity, discipline representation, and institutional size. After removing incomplete or invalid submissions (e.g., uniform responses, short completion times, or excessive missing data), 782 valid cases were retained. Participants spanned multiple academic domains, including engineering, humanities, social sciences, and the arts. The gender distribution was balanced (44.28% male, 55.72% female), and the sample covered all academic years (27.37% first-year, 45.01% second-year, and 23.21% third-year). The survey instrument was developed by adapting and synthesizing validated scales from prior empirical studies.

All items were measured using a 5-point Likert scale ranging from strongly disagree (1) to strongly agree (5). Platform-related variables—including system usability, content quality, and interface responsiveness—were derived from the End-User Computing Satisfaction (EUCS) model^1^and the Technology Acceptance Model (TAM)^2^, which continue to serve as validated foundations for satisfaction measurement in recent studies^4,5^. Meanwhile, psychological constructs such as emotional stability, subjective well-being, and self-regulation were incorporated from the educational psychology literature^7–9^. The final questionnaire comprised four thematic sections: (1) platform experience, (2) psychological well-being and trust, (3) satisfaction, and (4) demographic background. Data were collected via an online questionnaire distributed to students at multiple universities. After eliminating incomplete or invalid responses, 782 valid cases were retained for analysis. The sample displayed balanced gender representation (45% male, 55% female) and a diverse distribution of academic disciplines.

### Data preprocessing

Data preprocessing was conducted using Python and IBM SPSS 25. Categorical variables were transformed using one-hot encoding, while continuous variables were standardized via Z-score normalization to ensure comparability across different scales. Missing data were addressed through listwise deletion, and outliers were identified and removed based on boxplot inspection and Z-score thresholds. To mitigate multicollinearity, variance inflation factor (VIF) diagnostics were applied, and variables with excessive intercorrelations were excluded from further analysis. To address the class imbalance in the satisfaction outcome variable, particularly the underrepresentation of lower satisfaction categories, the Synthetic Minority Over-sampling Technique (SMOTE) was applied to the training dataset. This step was crucial for avoiding biased model predictions in minority classes, such as students reporting low satisfaction, which might otherwise be overlooked during model training. Finally, the dataset was randomly partitioned into training and test subsets using an 80:20 ratio to facilitate model evaluation on unseen data.

### Model construction and evaluation

Three machine learning models were evaluated to determine the optimal predictive algorithm: Logistic Regression (serving as a baseline linear model), Support Vector Machine (SVM), and Random Forest (RF). While SVM exhibited strong performance (accuracy = 0.8596, MSE = 0.1660), the RF model outperformed both alternatives by achieving the highest area under the ROC curve (AUC = 0.9775), indicating superior classification accuracy and discriminative capacity across satisfaction levels.

Given these results, Random Forest was selected as the primary modeling technique for its robustness with high-dimensional data and capacity to handle complex, nonlinear relationships. The model was implemented using Python’s sci-kit-learn library. A permutation-based feature importance analysis was conducted to enhance model transparency, enabling the ranking of variables based on their marginal contribution to prediction accuracy. In addition, Partial Dependence Plots (PDPs) were used to visualize the marginal effects of key features, thereby offering interpretive insight into the nonlinear interactions between platform attributes and psychological variables. This dual capacity for high predictive performance and intuitive interpretability renders Random Forest well-suited to complex educational contexts, where multifaceted, interdependent influences shape user satisfaction. It is an accurate classification tool and provides a valuable analytical lens for understanding how various factors collectively shape learner experiences.

### Ethical approval

This study received formal ethical approval from the Institutional Ethics Committee of Zhejiang Institute of Communications (Ref. number: REC/02/2018 [RZ-2018-07]). The research was deemed to pose no significant ethical risk and was conducted in accordance with the Declaration of Helsinki and institutional guidelines governing research involving human participants. Data was collected in March 2018 using an anonymous online questionnaire administered via the Wenjuanxing platform. The survey focused exclusively on non-sensitive psychological constructs, such as emotional stability and subjective well-being, and did not include physiological measures or personally identifiable information. Before participation, all respondents were informed of the study’s objectives, anonymity, and right to withdraw at any time. Informed consent was obtained from all participants before survey submission.

## Results

### Feature importance indicators

**Fig. 2:**
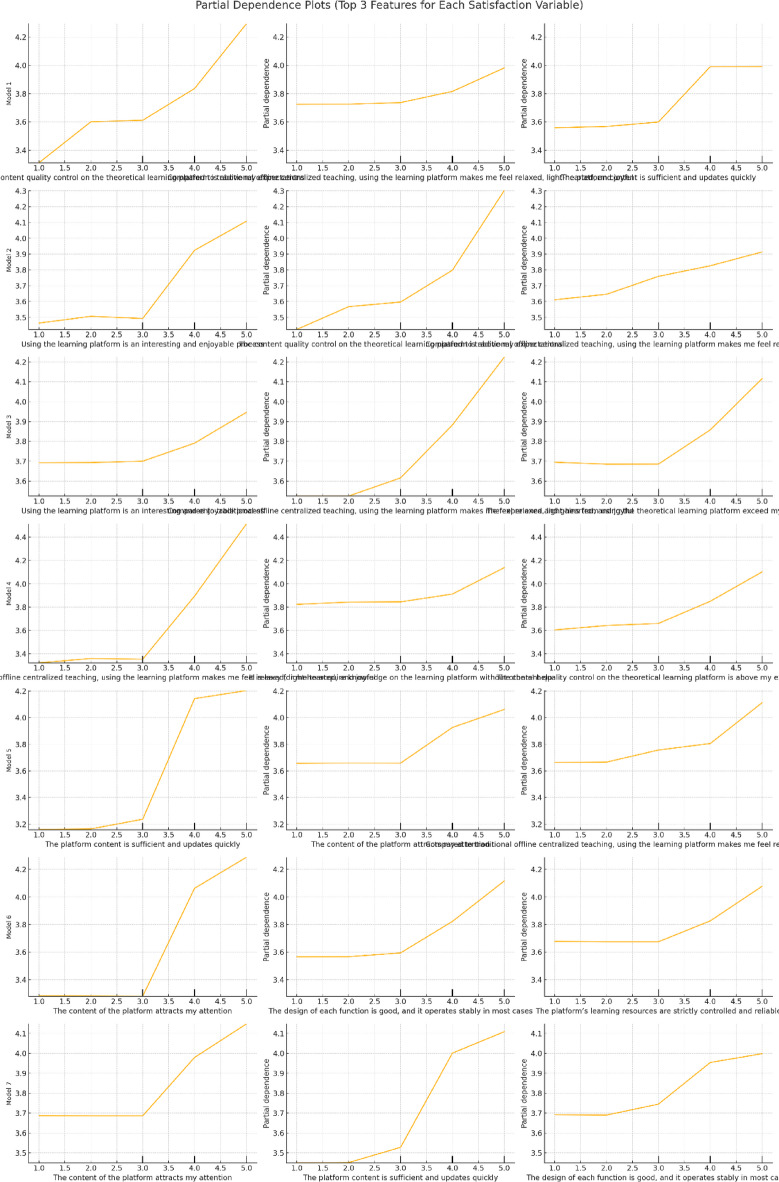
Partial dependence plots (PDPs) for the top 3 features of student satisfaction.

A permutation-based feature importance analysis was conducted using the Random Forest (RF) model to explore the nonlinear contributions of individual predictors to student satisfaction. Subsequently, the top three predictors were examined using Partial Dependency Plots (PDPs), which visualize the marginal effects of each variable on the model’s output (Fig. 2). Corresponding importance scores are detailed in Table 1.

The most influential variable—“The content quality control on the theoretical learning platform is above my expectations”—received the highest mean importance score (0.091). PDP indicates a pronounced threshold effect: satisfaction remains stable at low to moderate perceived content quality but increases sharply once quality surpasses a critical point. This pattern reflects an “expectation gap” phenomenon, where exceeding baseline expectations significantly enhances user satisfaction. The second-ranked feature—**“**Compared to traditional offline centralized teaching, using the learning platform makes me feel relaxed, light-hearted, and joyful**”**—demonstrated a saturation effect. Initial increases in emotional comfort were associated with substantial improvements in satisfaction, but this effect diminished as comfort levels continued to rise. This diminishing effect suggests diminishing marginal returns beyond a certain emotional threshold. The third key variable—**“**The platform content is sufficient and updates quickly**”**—exhibited a stepwise relationship. Satisfaction improved markedly when content sufficiency transitioned from low to moderate, but further gains plateaued at higher levels. This plateau effect indicates the presence of a “minimum quality standard” below which satisfaction is compromised but beyond which additional updates yield limited added value.

These findings underscore that student satisfaction is governed by complex, nonlinear dynamics rather than simple additive relationships. The presence of threshold, saturation, and stepwise effects highlights the utility of machine learning techniques, particularly Random Forest, for uncovering intricate variable interactions often missed by traditional linear approaches and motivates further investigation into how such nonlinearities influence overall model performance.

**Table 1:** Top 10 features ranked by permutation importance scores.

Feature	ImportanceMean	ImportanceStd
The content quality control on the theoretical learning platform is above my expectations	0.091481863	0.014855093
Compared to traditional offline centralized teaching, using the learning platform makes me feel relaxed, light-hearted, and joyful	0.043782175	0.004011027
The platform content is sufficient and updates quickly	0.033084084	0.00649016
Using the learning platform is an interesting and enjoyable process	0.031132518	0.007200103
I believe the learning platform is trustworthy	0.02669666	0.008337145
The experience and gains from using the theoretical learning platform exceed my expectations	0.024678244	0.004941811
It is easy for me to acquire knowledge on the learning platform without others’ help	0.017551052	0.010364457
The experience on the theoretical learning platform exceeds my pre-use expectations	0.015642794	0.004767496
The content of the platform attracts my attention	0.011245411	0.003579186
The design of each function is good, and it operates stably in most cases	0.008358677	0.004016783

### Correlation analysis

**Fig. 3:**
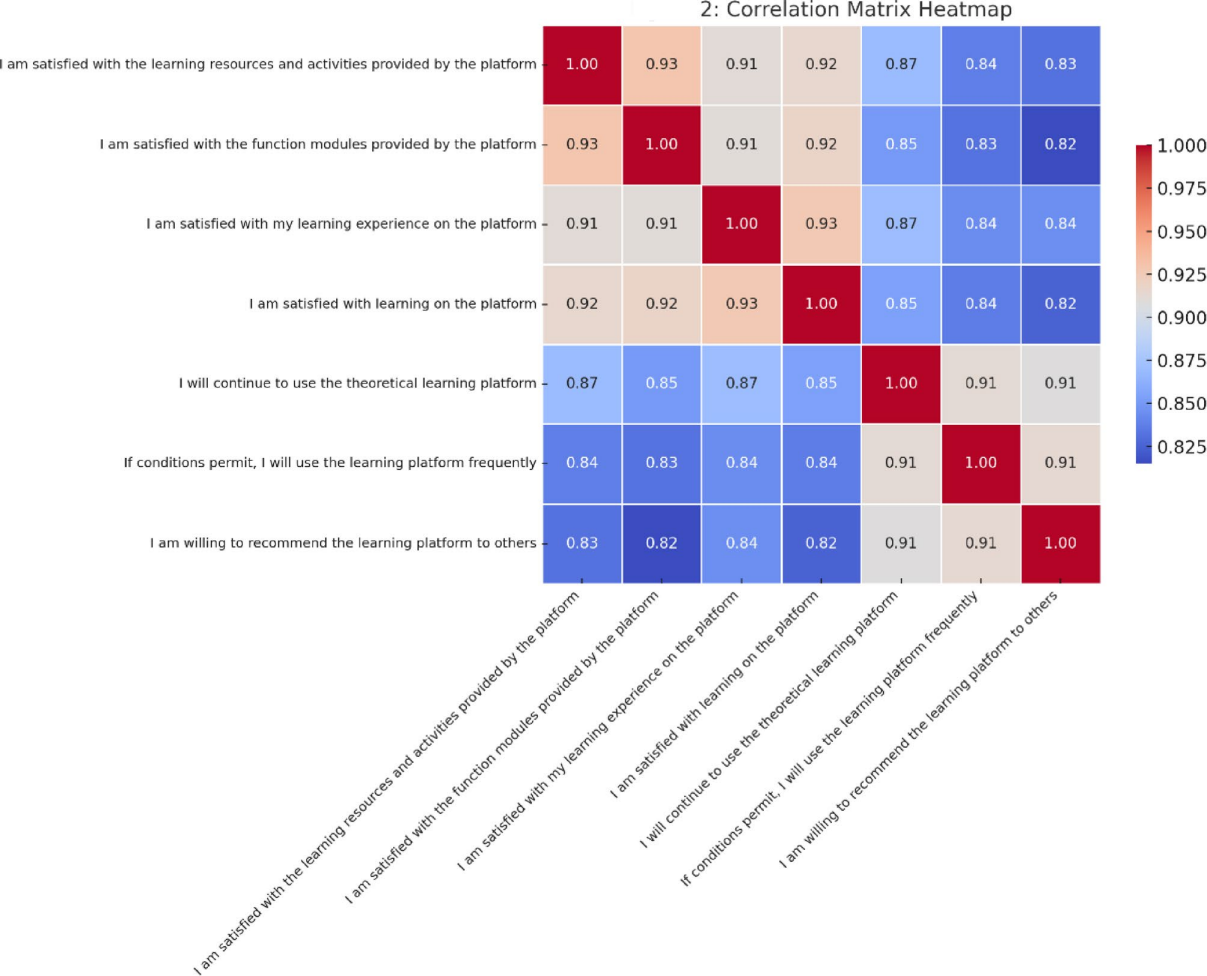
Correlation Matrix Heatmap of core satisfaction items.

A correlation matrix heatmap (Fig. 3) was generated to examine the internal consistency among satisfaction-related items. The analysis revealed consistently strong positive correlations across the seven core variables, indicating structural coherence within the satisfaction construct.

Notably, the strongest correlation was observed between **“**I am satisfied with my learning experience on the platform**”** and **“**I am satisfied with learning on the platform**”** (*r* = 0.93), suggesting semantic redundancy between general satisfaction indicators. Similarly, **“**I am satisfied with the function modules provided by the platform**”** and **“**I am satisfied with the learning resources and activities**”** also demonstrated high intercorrelation (*r* = 0.93), reflecting an integrated perception of content and functionality. In addition, behavioral intention items—such as willingness to continue using the platform and likelihood of recommending it to others—were strongly correlated with overall satisfaction measures (*r* > 0.90). This finding supports the established theoretical linkage between affective judgments and behavioral intentions, consistent with frameworks such as ECM and TAM (Bhattacherjee, 2001; Roca et al., 2006)^2,30^, and reaffirmed in recent research on continuance intention and platform satisfaction^4,5^.

These results validate the scale’s psychometric soundness and provide empirical justification for modeling satisfaction as a unidimensional composite index or a multidimensional latent construct, laying the foundation for subsequent predictive modeling strategies.

A comprehensive set of psychometric analyses was conducted to evaluate potential item redundancy and verify the construct validity of the satisfaction scale. The scale exhibited excellent internal consistency (Cronbach’s α = 0.925), and sampling adequacy was confirmed by a high Kaiser-Meyer-Olkin (KMO) value of 0.932. Additionally, Bartlett’s test of sphericity yielded statistically significant results (χ² = 7835.43, *p* < 0.001), indicating that the correlation matrix was factorable. Subsequent confirmatory factor analysis (CFA) supported the multidimensional nature of the construct, with model fit indices falling within acceptable ranges (CFI = 0.931, TLI = 0.912, RMSEA = 0.045). These findings indicate that the scale items capture multiple, distinguishable facets of user satisfaction, even with high inter-item correlations. The results mitigate concerns about semantic redundancy and substantiate the appropriateness of retaining all items for further analysis.

### Model performance evaluation

Classification and regression metrics, including accuracy, F1 score, and mean squared error (MSE), were employed to assess the Random Forest model’s predictive efficacy. As shown in Table 2, the model achieved a training accuracy of 95.8% and a test accuracy of 83.4%, demonstrating strong fitting and adequate generalization. Cross-validation yielded consistent results (accuracy = 93.9%).

**Table 2:** Performance evaluation.

Setting	Accuracy	Precision	Recall	F1-Score	MAP
Training Set	0.958	0.958	0.958	0.958	0.054
Test Set	0.834	0.840	0.834	0.836	0.223
Cross Validation	0.939	N/A	N/A	N/A	N/A

**Fig. 4:**
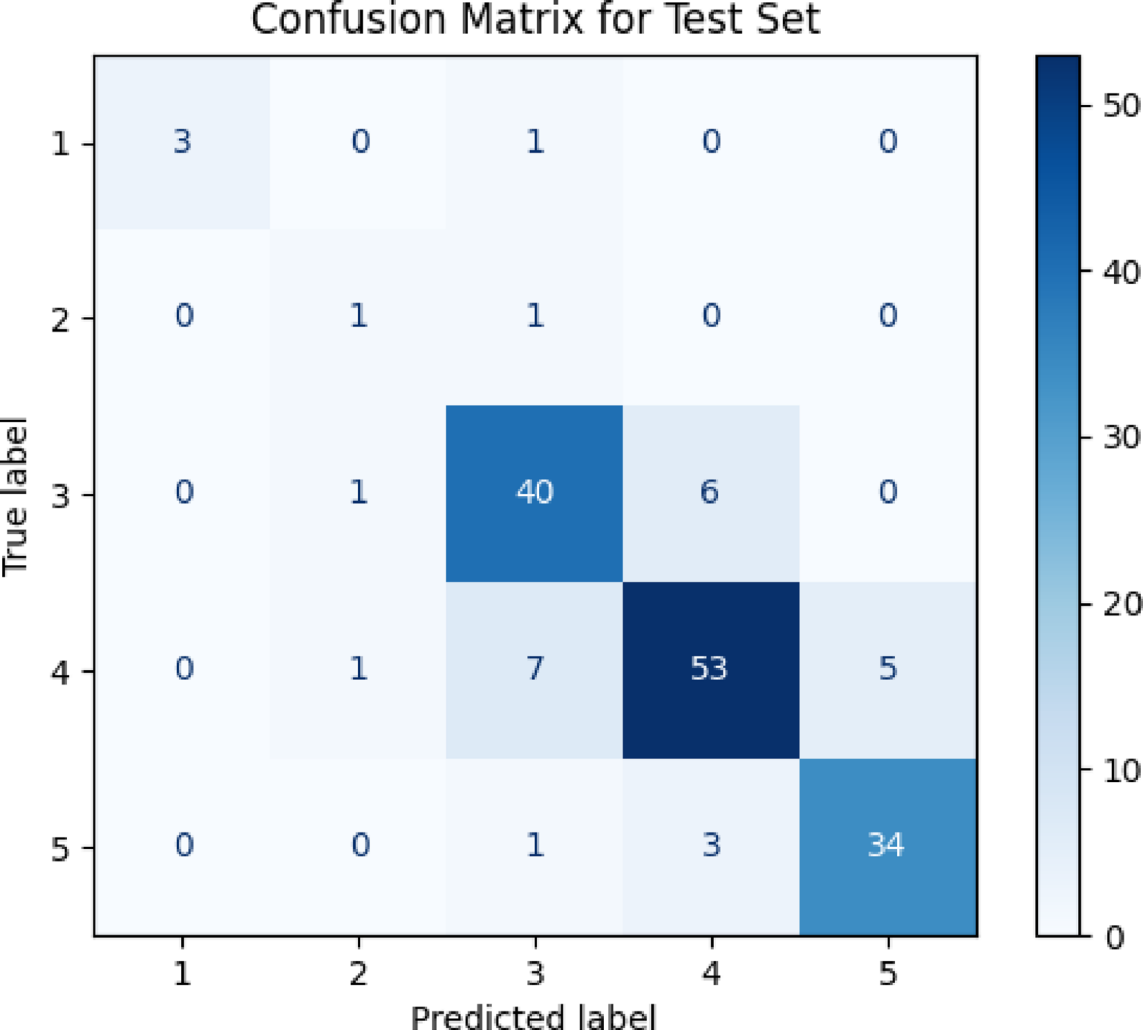
Confusion Matrix of Test Set Predictions.

The confusion matrix (Fig. 4) illustrates high classification accuracy for Categories 1, 3, and 5. However, Category 2 showed disproportionate misclassification, reflecting a performance shortfall due to class imbalance. This misclassification pattern highlights a well-known challenge in supervised classification, where infrequent categories, despite their theoretical importance, are often underrepresented and less reliably predicted. Despite this localized weakness in Category 2, Random Forest remains the most appropriate algorithm for this task due to its superior overall accuracy, robustness to noise, and ability to model high-dimensional, nonlinear data structures.

### ROC analysis

**Fig. 5:**
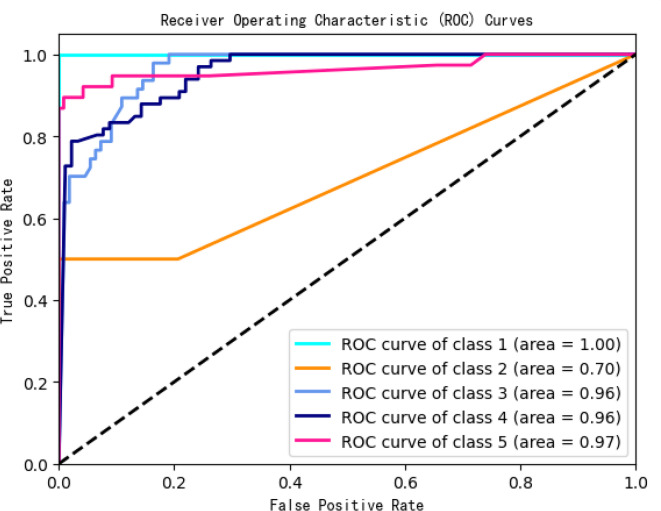
ROC Curve for satisfaction categories (1–5).

To further evaluate classification quality, Receiver Operating Characteristic (ROC) curves were plotted for all five satisfaction categories (Fig. 5). The model achieved AUC values > 0.95 for Categories 1, 3, and 5, indicating strong discriminatory power. In contrast, Category 2 recorded an AUC of 0.70, suggesting diminished classification reliability for this group. This discrepancy stems from severe class imbalance, as Category 2 constituted only 6% of the responses. The model’s limited exposure to this group’s feature distribution impaired its ability to generalize patterns effectively. Such imbalance skews overall accuracy and obscures meaningful variance in moderate satisfaction responses.

These findings underscore the need for targeted adjustments—such as oversampling, synthetic data generation, or enhanced algorithmic tuning—to address underrepresented classes and ensure fairness across satisfaction levels. These limitations directly motivate the optimization strategies proposed in the following section.

### Model optimization and future directions

While the Random Forest model demonstrated robust overall performance, its limited ability to accurately classify Category 2 suggests a critical area for refinement. Given that this limitation stems primarily from class imbalance, several optimization strategies are recommended. First, the sample size for underrepresented satisfaction levels could be increased through targeted data collection or synthetic oversampling techniques (e.g., SMOTE variants). Second, ensemble methods such as AdaBoost or XGBoost may be incorporated to enhance sensitivity to misclassified instances by iteratively reweighting weak learners. Finally, systematic hyperparameter tuning can further calibrate model complexity, reduce overfitting, and improve generalization capacity.

These strategies offer a comprehensive pathway for enhancing predictive accuracy and fairness, particularly in heterogeneous educational settings where satisfaction distributions may be uneven. Addressing these challenges is crucial for developing reliable, scalable, and inclusive satisfaction modeling frameworks. This study offers a robust and adaptable framework for satisfaction modeling in increasingly personalized digital learning environments by integrating psychological constructs with platform features and proactively addressing class imbalance.

## Discussion

### Key findings and practical implications

This study employed a Random Forest–based predictive framework to identify key determinants of university students’ satisfaction with online learning platforms. Among the various predictors, platform stability and content update frequency emerged as the most salient factors—reinforcing earlier findings^4,5,18^, while enhancing explanatory depth through nonlinear modeling. The model demonstrated robust classification performance, achieving AUC values above 0.96 for Categories 1, 3, and 5. However, its performance for Category 2 was notably lower (AUC = 0.70), likely due to data imbalance or the absence of latent moderating variables.

This discrepancy carries essential practical and theoretical implications. Moderate satisfaction levels may signal ambivalent or inconsistent user experiences, possibly driven by unmet expectations, fluctuating learning environments, or latent cognitive-emotional dynamics. It has been noted that such “design–reality gaps” arise when platform features fail to align with users’ goals, creating perceptual dissonance^31^. Such design–reality gaps emphasize the need for platform designers to go beyond optimizing technical attributes and address intermediate satisfaction states, which may serve as early indicators of disengagement. The results support a multi-layered intervention strategy. While ensuring system reliability and content freshness is foundational, these must be complemented by personalized recommendation engines, adaptive user interfaces, and sentiment-aware design. Early warning systems—enabled by real-time analytics—could help detect dissatisfaction trends and support timely pedagogical or technical interventions.

In addition, PDP analyses revealed nonlinear phenomena, including threshold and saturation effects. For instance, student satisfaction only increased substantially when perceived content quality exceeded a critical threshold, illustrating an “expectation gap.” Similarly, emotional comfort displayed diminishing returns beyond a certain point. These insights advocate for threshold-sensitive design approaches that aim not only to meet but to exceed baseline learner expectations, and motivate further investigation into how these nonlinearities influence classification reliability across satisfaction categories (see Sect."Conclusion").

### Comparative analysis and theoretical implications

Methodologically, this study extends the literature by illustrating how machine learning, specifically Random Forest, can complement and expand classical satisfaction models. While effective for testing predefined hypotheses, traditional logistic regression and structural equation modeling (SEM) techniques are inherently constrained by their linear and additive assumptions. Prior research^6^has noted such limitations, particularly in modeling complex interactions among psychological and contextual factors.

By contrast, the Random Forest approach employed here captured intricate nonlinear dynamics, such as threshold effects and interaction saturation, that are difficult to detect using conventional models. Building on descriptive frameworks^23^, this study moves beyond surface-level analysis by identifying influential predictors and explaining how their effects evolve across different satisfaction levels, offering insight into the mechanisms underlying satisfaction formation across diverse learner profiles. Integrating interpretability tools such as Partial Dependence Plots further enhances the explanatory transparency of the model, bridging the gap between prediction and interpretation.

Our findings align with studies that emphasize the superior predictive performance of ensemble methods in educational data contexts^24^. However, this study’s contribution extends beyond predictive accuracy. The model captures context-dependent interaction patterns that yield actionable insights for platform design, intervention targeting, and learner segmentation by embedding psychological constructs within a flexible, data-driven framework. Furthermore, the results corroborate psychological research demonstrating that emotional stability and self-regulation exert nonlinear, user-specific influences on satisfaction^7^. These findings challenge the additive assumptions embedded in models like TAM and ECM, underscoring the need for adaptive modeling strategies that reflect the cognitive-affective variability of digital learners.

Rather than positioning machine learning as a replacement for classical frameworks, we propose it as a methodological extension, enhancing explanatory precision and expanding the theoretical boundaries of satisfaction research in digital education. Classical models remain indispensable for theory testing and causal mechanism validation, particularly in hypothesis-driven inquiries. Combining traditional and data-driven approaches thus offers a more comprehensive toolkit for understanding and improving student experiences in online learning environments.

### Conclusion

Drawing on empirical data from 782 university students, this study demonstrates the methodological efficacy and theoretical relevance of employing a Random Forest–based framework to model satisfaction in online learning environments. Platform stability and content update frequency were identified as the most influential determinants among the predictors examined. More importantly, the machine learning approach enabled the detection of intricate nonlinear patterns—such as threshold effects, saturation curves, and discontinuous response behaviors—that are often masked by conventional linear modeling techniques.

Notwithstanding these contributions, the study is subject to several limitations. First, the sample was confined to Chinese vocational college students, which may limit the generalizability of findings across diverse educational systems and cultural settings. Second, although the model incorporated a range of technological and psychological predictors, it excluded several potentially salient constructs, including intrinsic motivation, grit, cognitive load, and social presence. Third, while Random Forests offer relative interpretability through permutation importance and partial dependence plots (PDPs), they do not provide the explicit causal mapping characteristic of traditional structural equation models.

To overcome these limitations, future studies should expand the sampling framework to encompass learners from different educational stages—such as primary, secondary, and adult education—and from varied cultural and institutional contexts, thereby enhancing external validity. Integrating broader psychological constructs could also provide a richer account of learner engagement and satisfaction. On the methodological front, the application of model-agnostic interpretability techniques such as SHAP (SHapley Additive exPlanations) and LIME (Local Interpretable Model-Agnostic Explanations) is recommended to facilitate more fine-grained, case-level interpretability and improve transparency in model reasoning. Additional avenues for refinement include improving the representation of minority satisfaction classes through targeted oversampling or synthetic augmentation (e.g., SMOTE), incorporating behavioral trace data such as session duration and clickstream activity, and experimenting with alternative ensemble learning methods (e.g., AdaBoost, XGBoost) to assess comparative performance. These methodological enhancements would support the development of more equitable, robust, and adaptable predictive models for educational technology applications.

Theoretically, the study challenges the linearity assumptions underpinning traditional frameworks such as the Technology Acceptance Model (TAM) and Expectation Confirmation Model (ECM), and advocates for adopting cognitively and affectively enriched, interaction-sensitive modeling paradigms. Practically, the findings offer actionable design implications for platform developers and institutional stakeholders, underscoring the importance of adaptive personalization, real-time feedback mechanisms, and threshold-aware interface strategies to optimize user satisfaction.

Although bounded by demographic scope and variable selection, this work advances the satisfaction modeling literature by introducing a hybrid, data-driven paradigm that bridges machine learning methodologies with established theoretical constructs. In doing so, it provides scalable, evidence-based recommendations for the design of inclusive, context-sensitive, and learner-centered digital education systems. Future studies are therefore encouraged to incorporate additional psychological variables—such as cognitive load and resilience—and to engage more diverse learner populations across educational stages and cultural settings, thereby enhancing generalizability and further supporting the development of inclusive, user-centered educational platforms.

## Electronic supplementary material

Below is the link to the electronic supplementary material.

Supplementary Material 1

Supplementary Material 2

Supplementary Material 3

## Data Availability

Data is provided within the manuscript or supplementary information files.
