# Supplementary material for: Modeling student satisfaction in online learning using random forest
Source: Sci Rep. 2025 Jul 2;15:23254. doi: 10.1038/s41598-025-06686-3 (PMC12222467; doi:10.1038/s41598-025-06686-3)
Supplement: Supplementary file 3 — Supplementary Material 3 [file 41598_2025_6686_MOESM3_ESM.docx]

**Response regarding the questionnaire**

We confirm that the questionnaire used in this study was developed by the authors, grounded in an extended Technology Acceptance Model (TAM) framework. While the overall structure and wording of the items were tailored to the context of smart learning platforms, each item was carefully adapted from well-established and validated prior studies to ensure content validity and theoretical consistency.

The constructs measured include perceived usefulness, ease of use, trust, confirmation, satisfaction, perceived enjoyment, platform quality, information quality, and continued use intention. Each construct was operationalized using three to four items, drawing from foundational works such as Davis (1989), Bhattacherjee (2001), DeLone and McLean (1992), among others. A complete mapping of constructs, item codes, item content, and source references is presented in Table 1.

Importantly, we confirm that **no proprietary third-party scales or copyrighted instruments were used**. All items were adapted from open-access or academic sources that do not require permission for reuse under the CC BY license. Therefore, **no formal permission document is required**.

**Table 1.** Questionnaire Item Design for Measuring Constructs in the TAM Framework

| **Construct** | **Item Code** | **Questionnaire Item** | **Source** |
| --- | --- | --- | --- |
| **Perceived Usefulness** | PU1 | Using the smart learning platform can improve my learning efficiency. | Bhattacherjee (2001)[1] |
|  | PU2 | Using the platform can improve the quality of my learning. |  |
|  | PU3 | I can find practical knowledge and information related to fundamental theory. |  |
|  | PU4 | The content of the platform is strictly controlled and very useful. |  |
| **Perceived Ease of Use** | PEU1 | It is easy for me to use the platform without external help. | Davis (1989)[2], Moon & Kim(2001)[3], Hong et al. (2006)[4] |
|  | PEU2 | The interactive interface of the platform is straightforward to understand. |  |
|  | PEU3 | It is very convenient to use the platform to learn relevant resources. |  |
|  | PEU4 | The platform is simple and easy to use, with fast operation. |  |
| **Perceived Trust** | PT1 | The platform is trustworthy. | Tan (2001)[5] |
|  | PT2 | I believe the platform will not leak my private information. |  |
|  | PT3 | The learning materials provided are authoritative and reliable. |  |
| **Confirmation** | CON1 | The experience and gains of using the platform to learn exceed my expectations. | Bhattacherjee (2001)[1] |
|  | CON2 | The platform experience is higher than expected before using it. |  |
|  | CON3 | The content and quality control surpass my initial expectations. |  |
| **Satisfaction** | SAT1 | I am satisfied with the learning resources and activities provided by the platform. | Bhattacherjee (2001) ^[1]^, Oliver (1980)[6] |
|  | SAT2 | I am satisfied with the functional modules of the platform. |  |
|  | SAT3 | I am satisfied with the learning experience on the platform. |  |
|  | SAT4 | Overall, I am delighted with my use of the platform. |  |
| **Perceived Enjoyment** | PE1 | Using the platform makes me feel more relaxed, learn efficiently, and be happy. | Davis (1989)[1], Tsang (2004)[7] |
|  | PE2 | The platform offers exciting content like micro-videos, case studies, and e-books. |  |
|  | PE3 | Using the platform is an enjoyable and exciting process. |  |
| **Platform Quality** | SYS1 | The platform's response speed is fast, allowing smooth use. | DeLone & McLean (1992)[8] |
|  | SYS2 | Each function's design is perfect and stable in operation. |  |
|  | SYS3 | The interface layout is user-friendly and easy to use for novices. |  |
| **Information Quality** | INF1 | The platform provides sufficient content with quick updates. | DeLone & McLean (1992)[8] |
|  | INF2 | The platform's learning resources are strictly controlled and reliable. |  |
|  | INF3 | The platform's content attracts my attention. |  |
| **Continued Use Intention** | CI1 | I will continue to use the platform to study. | Bhattacherjee (2003)[1] |
|  | CI2 | If possible, I will frequently use the platform. |  |
|  | CI3 | I will recommend the platform to others. |  |

Reference

1. Bhattacharjee, A. An empirical analysis of the antecedents of electronic commerce service continuance [J].Decision support systems,2001, 32(2):201–214.

2. Davis, F. D. (1989). Perceived usefulness, perceived ease of use, and user acceptance of information technology. MIS Quarterly, 13(3), 319–340.

3. Moon, J.-W., & Kim, Y.-G. (2001). Extending the TAM for a world-wide-web context. Information & Management, 38(4), 217-230.

4. Hong, S., Thong, J. Y., & Tam, K. Y. (2006). Understanding continued information technology usage behavior: A comparison of three models in the context of mobile internet. Decision support systems, 42(3), 1819-1834.

5. Tan Y.H., & Thoen W.(2001). The Role Of Trust In Electronic Commerce.Electronic Markets11(1),15–25.

6. Oliver, R. L. (1980). A cognitive model of the antecedents and consequences of satisfaction decisions. Journal of marketing research, 17(4), 460-469.

7. Tsang, M. M. (2004). Consumer attitudes toward mobile advertising: An empirical study. International Journal of Electronic Commerce, 8(3), 65–78.

8. DeLone, W. H., & McLean, E. R. (1992). Information systems success: The quest for the dependent variable. Information Systems Research, 3(1), 60–95.
